# A systematic review of workplace triggers of emotions in the healthcare environment, the emotions experienced, and the impact on patient safety

**DOI:** 10.1186/s12913-024-11011-1

**Published:** 2024-05-09

**Authors:** Raabia Sattar, Rebecca Lawton, Gillian Janes, Mai Elshehaly, Jane Heyhoe, Isabel Hague, Chloe Grindey

**Affiliations:** 1 Bradford Teaching Hospitals NHS Foundation Trust, Bradford, BD9 6RJ UK; 2 Anglia Ruskin University, Cambridge, CB1, 1PT UK; 3 University of London, London, EC1V 0HB UK

**Keywords:** Emotions, Healthcare work environment, Patient safety, Systematic review

## Abstract

**Background:**

Healthcare staff deliver patient care in emotionally charged settings and experience a wide range of emotions as part of their work. These emotions and emotional contexts can impact the quality and safety of care. Despite the growing acknowledgement of the important role of emotion, we know very little about what triggers emotion within healthcare environments or the impact this has on patient safety.

**Objective:**

To systematically review studies to explore the workplace triggers of emotions within the healthcare environment, the emotions experienced in response to these triggers, and the impact of triggers and emotions on patient safety.

**Methods:**

Following the Preferred Reporting Items for Systematic Reviews and Meta-Analyses guidelines, four electronic databases were searched (MEDLINE, PsychInfo, Scopus, and CINAHL) to identify relevant literature. Studies were then selected and data synthesized in two stages. A quality assessment of the included studies at stage 2 was undertaken.

**Results:**

In *stage 1*, 90 studies were included from which seven categories of triggers of emotions in the healthcare work environment were identified, namely: patient and family factors, patient safety events and their repercussions, workplace toxicity, traumatic events, work overload, team working and lack of supervisory support. Specific emotions experienced in response to these triggers (e.g., frustration, guilt, anxiety) were then categorised into four types: immediate, feeling states, reflective, and longer-term emotional sequelae. In *stage 2*, 13 studies that explored the impact of triggers or emotions on patient safety processes/outcomes were included.

**Conclusion:**

The various triggers of emotion and the types of emotion experienced that have been identified in this review can be used as a framework for further work examining the role of emotion in patient safety. The findings from this review suggest that certain types of emotions (including fear, anger, and guilt) were more frequently experienced in response to particular categories of triggers and that healthcare staff's experiences of negative emotions can have negative effects on patient care, and ultimately, patient safety. This provides a basis for developing and tailoring strategies, interventions, and support mechanisms for dealing with and regulating emotions in the healthcare work environment.

**Supplementary Information:**

The online version contains supplementary material available at 10.1186/s12913-024-11011-1.

## Background

Healthcare is delivered in emotionally charged settings [1]. Worried patients present with complex health issues, and anxious relatives need information and support, and safe care is reliant on clinical judgement and effective multi-disciplinary teamwork within a time-pressured resource-limited, complex system. Working in this environment, healthcare staff experience a range of emotions (e.g., anxiety, anger, joy, sadness, pride, and guilt) which can impact the safety of the care delivered [1–5]. Clinical judgement often involves weighing up risk based on incomplete information and uncertain outcomes. Research outside healthcare [6–9] suggests that if, while making these decisions, healthcare staff experience strong emotions, this can influence their decisions and behavior.

Research focusing on the role of emotion in patient safety is still limited [1, 2, 10] and fragmented [11]. This may in part be because emotion research is complex. For example, the experience and influence of emotion can be approached and interpreted from a range of perspectives including cognitive and social psychology, cognitive neuroscience, and sociology. There is also a lack of consensus on what is meant by ‘emotion’. In decision-making research, ‘emotion’ has been distinguished from ‘affect’ [11]. In response to stimuli or situations, ‘emotion’ is viewed as a slower, more reflective process, whilst ‘affect’ is an instantaneous and automatic reaction. Other research has focused on identifying and examining different types of affect, such as ‘anticipatory affect’—an immediate, strong visceral state in response to stimuli e.g. anger (Knutson, 2008) and ‘anticipated affect’ – considering how current actions might make you feel in the future e.g. regret [12].

Research often lacks a clear distinction between the different types of feeling states being examined, and as such, it is difficult to build robust evidence of the processes involved and the role they each play in judgement and associated behaviour [11]. Furthermore, the emotions experienced by healthcare staff can be both positive and negative and can influence the delivery of safe care in positive and negative ways. Until more recently, the focus has tended to be on the impact of negative emotions, including their role in diagnostic accuracy [13, 14], time spent on history taking, examinations, and treatment decisions [15, 16], and the instigation of verbal checking during procedures [4]. More attention is now being given to the role of positive emotions in the workplace such as their effect on reasoning [17], and engagement and teamwork [18].

There are many potential triggers (e.g. physical, circumstantial, tangible, and psycho-social aspects of the immediate clinical work environment and the broader organisation) that generate a feeling state via reactions or interactions of emotion in the workplace. Research exploring some of the triggers of emotions within a healthcare environment has found that involvement in care that has gone wrong [4], and interactions with patients can elicit negative emotions [13–16] and that triggers of emotion can be at a clinical, hospital and system level [15]. Only a limited number of these studies have also explored how the emotions experienced by healthcare staff impact patient care [15, 16]. While emotion has a direct effect on patient care, it can also indirectly influence patient safety. Burnout, sickness absence, and turnover are impacted by emotion [19–21] and, in turn, are associated with healthcare organisations’ ability to provide safe care [21–23]. Due to the multifaceted approaches to research in this area, it is currently unclear what contexts and settings elicit emotions in healthcare staff, how these make healthcare staff feel, and the influence these feelings may have on decisions and actions relevant to providing safe patient care. There is therefore a need to synthesise the current evidence to help develop an in-depth understanding of the triggers of emotions experienced by healthcare staff in the work environment, the emotions experienced and the impact these may have on patient safety.

## Methods

The protocol was pre-registered on Prospero (ID: CRD42021298970).

### Aims

This systematic review aimed to identify gaps in the evidence by answering these research questions:

1. What triggers emotions in the healthcare work environment?
2. What are the emotions experienced in response to these triggers?
3. Are certain emotions more often experienced in the context of particular triggers?
4. What impact do different triggers/ emotions have on patient safety processes and outcomes?

### Search strategy and databases

Four electronic databases (MEDLINE, PsychInfo, Scopus, and CINAHL)( were systematically searched in March 2020 and updated in January 2022. Only studies published since 2000 were sought as this was when the Institute of Medicine’s seminal report, ‘To Err is Human’’ [24] was published promoting a widespread focus on patient safety. The search strategy had three main foci (patient safety, emotions, and healthcare staff). Previous systematic reviews examining any of these topics in combination; patient safety [25] and healthcare staff [26]were used to guide search strategy development. As a foundation to develop the search terms in relation to emotions, the six basic emotions (fear, anger, joy, sadness, disgust, and surprise) described by Ekman [27] were included, with synonyms for emotion. This resulted in a search strategy that combined all three concepts (Available in Appendix 1). The reference lists of all included studies were hand-searched.

### Eligibility criteria

Studies were included if they were: published post-2000, original empirical research (either quantitative, qualitative, or mixed-methods), published in English, conducted in any healthcare environment, and included healthcare staff as participants. Studies were excluded if they; focused on healthcare staff’s non-work related emotions, included healthcare students/staff who were not involved in direct patient care (e.g. administrative staff), or if the primary focus was on longer-term emotional states (e.g. burnout and emotional exhaustion) with no reference to specific emotions.

1. This review had two stages:
2. Stage 1: The first stage addressed the first three research questions and identified studies focused on triggers of emotions in the healthcare work environment and the specific emotions experienced by healthcare staff in response to these.
3. Stage 2: The second stage examined the fourth research question and identified the impact of triggers and/or emotions on patient safety outcomes and processes. The studies included in stage 1 were further screened and considered at this stage if they included either (i.) triggers of emotions and their relationship with patient safety, (ii.) emotions experienced and their relationship with patient safety outcomes or processes (iii.) triggers, emotions and the relationship with patient safety outcomes or processes.

### Study selection

PRISMA guidelines [28] for study selection were followed. The study selection process is described below. Throughout each stage, all decisions and any uncertainty or discrepancies were discussed by the review team to achieve consensus.

Stage 1: Title and abstract then full-text screening, was conducted by IH & CG independently and then discussed together. RL reviewed a random 10% at the abstract review stage and all included full-text articles.

Stage 2: RS independently conducted abstract and title screening for all included studies. A random 10% of these were each independently screened by two reviewers (JH&RL). Full texts were obtained for all studies deemed potentially eligible for inclusion. All full texts were screened by RS. JH &RL double-screened half each. A final set of studies meeting all the eligibility criteria was identified for data extraction.

Assessment of the methodological quality of included studies was carried out using the 16-item quality assessment tool (QuADS) [25] which is appropriate for studies using different methodological approaches. Quality assessment was undertaken independently for two studies by three reviewers (RS, GJ&JH) and scores were discussed to check for consistency. RS&GJ completed a quality assessment for the remaining studies and discussed scores to check for consistency. No studies were discarded based on low scoring.

### Data extraction

Stage 1: A data extraction form developed in Microsoft Excel by IH&CG and agreed with the wider review team was used to extract: the study title, triggers of emotions in the healthcare work environment, and emotions experienced in response to these triggers. Two reviewers extracted these data (IH&CG), conferring at intervals throughout the extraction process to ensure consistency. Due to the large number of studies at this stage and our aim to take a broader approach to explore triggers and the associated emotions, we did not extract data related to study characteristics. We categorised both the types of triggers and emotions (drawing on existing theory and wider team expertise) to advance knowledge by providing an initial framework for further testing. The detailed process for categorisation of the emotions and triggers is described in supplementary Appendix 2.

Stage 2: A data extraction form developed and agreed upon by authors was used to extract: information on the study population, setting, design and methods used, key findings, conclusions, recommendations, triggers of emotions, emotions experienced, and impact on patient safety. CG&IH completed data extraction for included studies. This was cross-checked by RS and discussed with all reviewers.

### Categorising the patient safety outcomes and processes

The wide range of patient safety processes and outcomes (*n* = 50) from the included studies, meant it was necessary to reduce the data. Therefore, categories of outcomes were developed to allow the relationship between triggers/emotions and patient safety to be explored. The first step in the categorisation process involved a team of 8 patient safety researchers using a sorting process in which they were provided with 50 cards each describing a patient safety process/outcome extracted from the studies Working independently they grouped these 50 cards and gave each group a title. A large group discussion with all 8 patient safety researchers followed this, resulting in 7 categories. We then presented these categories and the items each contained, to a large group of patient safety researchers, healthcare staff, and patients (*n* = 16), at an inter-disciplinary meeting. This resulted in a final set of five categories representing patient safety processes: altered interaction with patients, disengagement with the job, negative consequences for work performance, defensive practice, being more cautious, negative impact on team relationships, and reduced staff confidence (see appendix 2 for further detail) and the sixth, patient safety outcomes.

## Results

### Quality assessment

There was a very high level of agreement between RS & GJ regarding the quality assessment. The quality of studies was variable, with total scores ranging from 79 to 48% across the studies. There was limited discussion of relevant theories related to emotions and patient safety, and few studies provided a rationale for the choice of data collection tools. There was also limited evidence to suggest stakeholders had been considered in the research design and limited – or often no justification for analytical methods used. A detailed quality assessment table is available in Appendix 3.

**Fig. 1:**
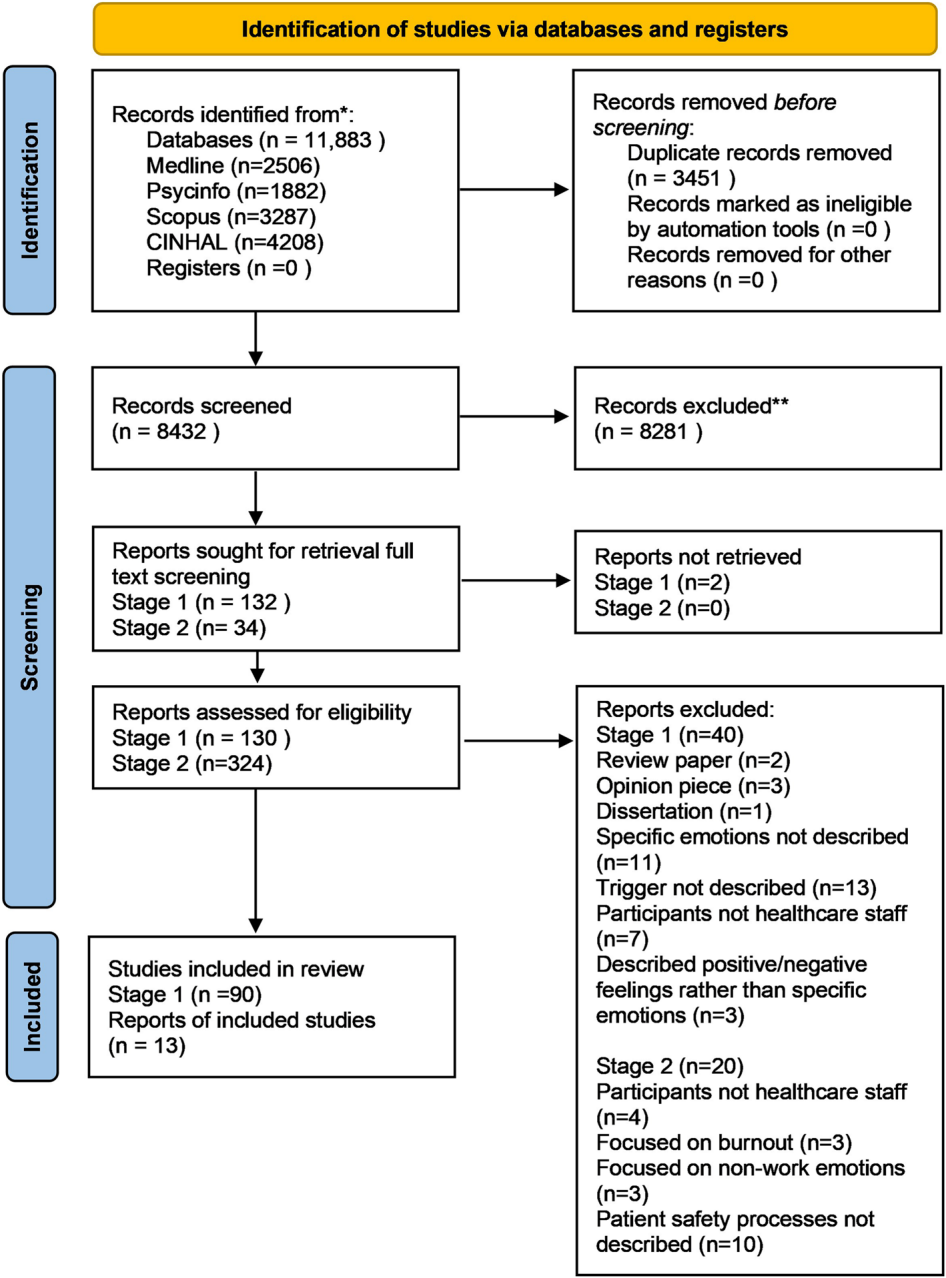
PRISMA flow diagram

After duplicates were removed; the search resulted in 8,432 articles for initial review which were downloaded into the reference management software Endnote (see PRISMA flow diagram in (Fig. 1). Stage 1: 90 studies met the inclusion criteria, investigating triggers of emotions in the healthcare work environment and the emotions experienced by healthcare staff in response to these.

Research question 1: What triggers emotions in the healthcare work environment?

The following categories of triggers were identified:

- (1) Patient and family factors ( included patient aggression, challenging patient behaviours, patient violence, patient hostility, and interactions with patients family)*
- (2) Patient safety events and their repercussions (including adverse events, errors, medical errors, and surgical complications)*
- (3) Workplace toxicity (including workplace bullying, and staff hostility)*
- (4) Traumatic events with negative outcomes for patients (including patient deaths/suicide, patient deterioration, and critical incidents).
- (5) Work overload (including work pressures and poor staffing levels).
- (6) Team working and lack of supervisory support (including teamwork and the lack of appropriate managerial support).

*The most common triggers investigated and reported in the literature

Research question 2: What are the emotions experienced in response to these triggers?

In response to the triggers described above, healthcare staff experienced four main types ofemotions:

- 1. Immediate: an instantaneous, visceral emotional response to a trigger (*e.g. fear, anxiety, anger, comfort, satisfaction, joy*).
- 2. Feeling states: short-lived, more mindful and conscious cognitive-based responses to a trigger (e.g. include feeling disoriented, confused, helpless, inadequate, alone).
- 3. Reflective/self-conscious: Mindful and conscious cognitive-based response after exposure to a trigger and following time to reflect on how others may perceive them (e.g. moral distress, guilt, pride).
- 4. Sequelae: chronic and longer-term mental health states that arise as a result of repeated exposure to a trigger and experiencing the emotions in response to that trigger over time (e.g. chronic depression, fatigue, distress, PTSD symptoms).

Research question 3: Are certain emotions more often experienced in the context of particular triggers?

**Fig. 2:**
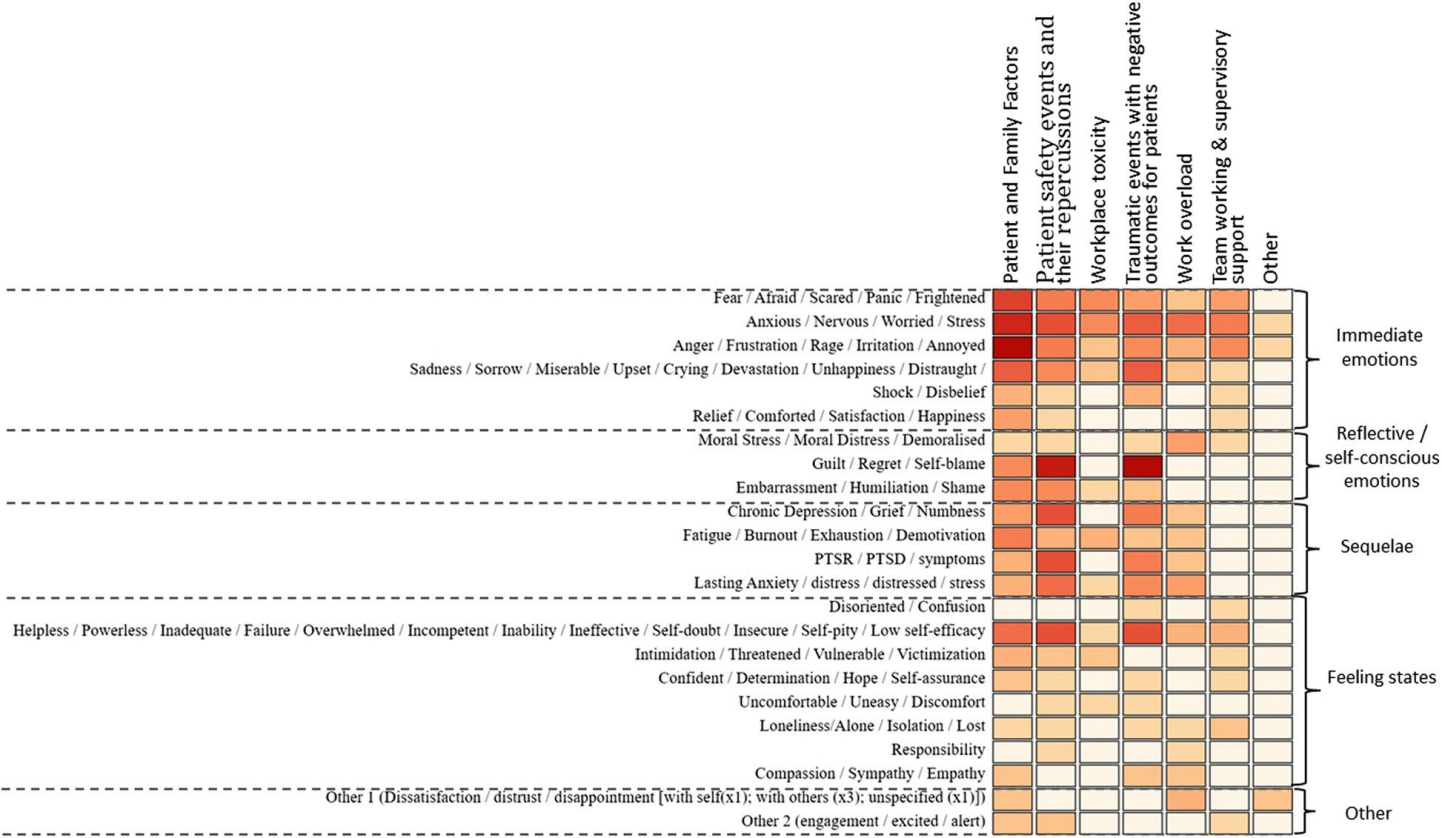
A heat map displaying the triggers of emotions experienced by healthcare staff and the emotions experienced in response to these. (The darker the colour on the heat map represents a higher frequency of that emotion being experienced across the studies)

The frequency of the emotions experienced across the studies in response to the categories of triggers is illustrated using a heat map (Fig. 2) developed by a data visualisation expert (MA). Below is a summary of the most frequently experienced emotions by healthcare staff in response to the categories of triggers across the studies.

- Patient and family factors: Immediate emotional responses including most commonly anger, frustration, etc.
- Patient safety events & their repercussions: Reflective/self–conscious emotions including guilt and regret
- Workplace toxicity: Immediate emotional responses including fear and anxiety
- Traumatic events with negative outcomes for patients: Reflective/self–conscious emotions including guilt and regret
- Work overload: Immediate emotional responses including anxiety and worry
- Team working & supervisory support: Immediate emotional responses including anxiety and worry

Stage 2 Research question 4. What impact do different triggers/ emotions have on patient safety processes or outcomes?

Thirteen publications [15, 16, 30–40] addressed this research question and were included at this stage.

All 13 studies [15, 16, 30–40] described the following patient safety processes/outcomes as being impacted by either the triggers of emotions or the emotions experienced; altered interaction with patients, disengagement with the job, negative consequences for work performance, defensive practice, being more cautious, negative impact on team relationships and reduced staff confidence and patient safety outcomes.

Depending on the nature of the studies, some explored only one of the patient safety processes/outcomes, whereas others focused on several. Eight studies used quantitative methods [30–37], three qualitative methods [15, 38, 39], and two mixed-methods designs [16, 40] to explore these relationships. Twelve studies were conducted in hospital settings [15, 16, 30–39], and one was conducted in hospital and community healthcare settings [40].

### The impact of triggers of emotion on patient safety processes or outcomes

Of the 13 studies [15, 16, 30–40], 10 [30–36, 38–40] included an exploration of or commented on the impact of triggers of emotion on patient safety processes/outcomes. None had a direct focus on exploring the relationship between the triggers and patient safety processes/outcomes; rather some stated this as one of multiple aims, whereas others reported any associations as part of the broader study findings. The relationship between specific triggers and patient safety processes/outcomes is displayed in Table 1.

**Table 1:** The relationship between triggers of emotion and patient safety process/outcomes

Study authors, year, country	Study design	Study population	Triggers of emotions	Patient safety outcomes	Disengagement with job	Negative consequences for work performance	Defensive practice	Being more cautious	Negative impact on team relationships
**Al Omar et al.** [30] **Saudi Arabia**	Cross-sectional (surveys)	*Nurses, physicians & pharmacists*	*Workplace bullying*	**X**	**X**	**X**			**X**
**Chambers et al.** [40] **New Zealand**	Cross-sectional (surveys and qualitative data)	*Healthcare staff from a range of medical specialities including anaesthesia, emergency medicine, paediatrics*	*Workplace bullying*		**X**	**X**	**X**		
**Chard et al.** [31] **USA**	Cross-sectional (surveys)	*Nurses*	*Medical errors*		**X**			**X**	
**Biggs et al.** [32] **UK**	Cross-sectional (surveys)	*Operating surgeons from a range of specialties*	*Surgical complications*					**X**	
**Bari et al.** [33] **Pakistan**	Cross-sectional (surveys)	*Pediatric residents*	*Medical errors*				**X**	**X**	
**Yildirim et al.** [34] **Turkey**	Cross-sectional (surveys)	*Nurses*	*Workplace bullying*		**X**	**X**		**X**	**X**
**Pinto et al.** [38] **UK**	Qualitative (interviews)	*General or Vascular surgeons*	*Surgical complications*				**X**		
**Hassankhani et al.** [39] **Iran**	Qualitative (interviews)	*Nurses*	*Workplace violence*		**X**				**X**
**Karga et al.** [35] **Greece**	Cross-sectional (surveys)	*Nurses*	*Medical errors*				**X**	**X**	
**Vanhaecht et al.** [36] **Netherlands**	Cross-sectional (surveys)	*Doctors and nurses*	*Patient safety incidents*			**X**	**X**		**X**

An *‘X’* in the patient safety processes/outcomes columns signifies that there is an association between these and the triggers of emotion

The most commonly described patient safety processes/outcomes following exposure to the triggers were 'disengagement with the job' [30, 31, 35, 40, 41] and ‘being more cautious’ [31–35]. There was increased disengagement with the job after experiencing workplace bullying, medical errors, and workplace violence. This included dissatisfaction and a desire to change jobs or leave healthcare practice. Involvement in medical errors, surgical complications, and workplace bullying also resulted in staff being more cautious. For example, they reported paying more attention to detail, keeping better patient records, and increased information-seeking from colleagues.

Within four studies, triggers were described as having a ‘negative impact on team relationships’ [30, 34, 36, 39] where being exposed to workplace bullying resulted in communication problems amongst staff and conflicts with co-workers. In one case being involved in a patient safety incident resulted in staff feeling uncomfortable within their team [36]. Workplace bullying and being involved in a patient safety incident also resulted in ‘Negative consequences for work performance’ in four studies [30, 34, 36, 40] which included delays in care delivery and being unable to provide quality care.

Exposure to triggers was also linked to ‘defensive practice’ [29, 32, 36]. There was an increase in defensive practice (e.g. ordering more tests, keeping errors to self, and avoiding risks) as a result of triggers such as workplace bullying and medical errors. 'Patient safety outcomes' were only described in one study, where it was perceived that there may be an increased risk of patient safety incidents such as increased medical errors, patient falls, adverse events, or patient mortality as a result of experiencing workplace bullying [30].

### The impact of emotions on patient safety processes or outcomes

Only three studies [15, 16, 37] included an exploration of the impact of emotions on either patient safety processes [15, 16] or outcomes [37]. These studies directly explored the relationship between the emotions experienced in response to triggers and patient safety processes/outcomes (see Table 2).

**Table 2:** The relationship between emotions experienced in response to triggers and patient safety processes and outcomes

Study authors, year, country	Study design	Study population	Triggers of emotions	Emotions experienced	Patient safety outcomes	Negative consequences for work performance	Defensive practice	Altered interaction with patients
**Isbell et al.** [16] **USA**	Cross-sectional (surveys using both quantitative & qualitative data)	*Nurses and physicians within Emergency departments*	*Emotionally evocative patients*	*Happiness, satisfaction, anger, frustration, irritation*		**X**	**X**	**X**
**Jalil et al.** [37] **UK**	Cross-sectional (surveys)	*Nurses within mental health departments*	*Patient aggression*	*Fear, guilt, sadness, anger*	**X**			
**Isbell et al.** [15] **USA**	Qualitative (interviews)	*Nurses and physicians within Emergency departments*	*Patient factors, hospital factors, system-level causes*	*Guilt, fear, frustration, anger, sadness, gratification, pride and happiness*		**X**		**X**

An ‘X’ in the patient safety processes/outcomes columns signifies that there is an association between these and the emotions experienced in response to triggers

In response to the emotions experienced by healthcare staff, ‘negative consequences for work performance’ were described[15, 16} as staff feeling unable to provide quality care or a delay or failure to provide appropriate examination/treatment was reported. Emotions also influenced defensive practices [16] such as risk avoidance, and provision of unnecessary treatment, and emotions were described as an influencing factor for overprescribing. Emotions were also found to influence physical restraint in mental health settings, where a positive correlation was reported between staff experiencing anger (as a result of patient aggression) and the approval of physical restraint [37].

The type of patients also influenced the emotions experienced by staff, which in turn altered the interaction with patients [15, 16]. Isbell et al. [16] found that encounters with angry and mental health patients elicited highly negative emotions such as fear and frustration, where staff spent less time with the patient and acted less compassionately. Increased interaction including expediting patient care and spending more time with the patient was associated with encounters with positive patients who elicited positive emotions (happiness, satisfaction)in staff. Isbell et al. [15] also found that patients with psychiatric conditions elicited negative emotions, which resulted in reduced patient interaction and potential for diagnostic error.

Whilst these studies [15, 16, 37] highlight that emotions may impact patient safety processes and/or outcomes, it was not always possible to ascertain the impact of specific emotions. Studies by Isbell et al. [15, 16] illustrate how negative emotions elicited by patients have a negative impact on patient safety processes, whereas positive emotions resulting from patient behaviours have the potential to enhance patient care. However, it is difficult to disentangle the effect of specific emotions, due to a lack of evidence regarding the link between individual emotions and patient safety processes and/or outcomes as studies have not attempted to explore this. Only Jalil et al. [37] focused specifically on anger as an emotion and its impact on restraint practices, where higher levels of anger were correlated with greater approval of restraint of mental health patients.

## Discussion

### Summary of main aims and findings

This review has identified and categorised the triggers of emotions in the healthcare work environment and the types of emotions experienced by healthcare staff in response to those triggers. It has also established the types of emotions more often experienced in the context of particular triggers, and the impact that different triggers and emotions may have on patient safety processes or outcomes. The most frequently reported triggers within the literature were *'patient and family factors', 'patient safety events and their repercussions',* and *‘workplace toxicity’*, and the most frequently cited emotions were ‘*anger, frustration, rage, irritation, annoyance’* and ‘*guilt, regret and self-blame’*. These emotions were all negative in nature, which may reflect a bias in the research literature.

The studies that focused on the triggers did not directly set out to assess the impact of triggers of emotions on patient safety processes or outcomes, but the reporting of this link in study findings did enable knowledge to be gained about this. Studies that did focus on emotions and patient safety directly explored the relationship between the emotions experienced in response to triggers and patient safety processes and outcomes. Previous literature [41–43] supports the link between the categories of patient safety processes identified within this review (*including reduced staff confidence, disengagement with the job, and defensive practice*) and patient care and or/patient safety, suggesting these processes may serve as mechanisms to influence patient safety. Only three studies were identified that focused on the impact of emotions experienced by healthcare staff within the work environment on patient safety processes/outcomes [15, 16, 37]. These studies highlight that a majority of emotional responses experienced by healthcare staff are negative and have the potential to result in negative work performance (*including being unable to provide quality care*), increased defensive practice, and negative patient safety outcomes *(increased approval of physical restraint*). In only one study [15], positive emotions were reported which resulted in positive outcomes including expediting patient care and spending more time with the patient.

The findings of this review support previous calls to acknowledge the importance of emotions and their impact on safe care [1, 2, 4, 5, 44], however, research in this area is still limited and fragmented [15, 16]. Except for one study [41], it was not possible to ascertain the association between specific emotions and patient safety processes, and even for this study (a cross-sectional survey), causal relationships were not demonstrated. Nevertheless, the findings do suggest that negative emotions elicited by patients within healthcare staff have a negative impact on the described patient safety processes and positive emotions have a positive impact on these processes. Earlier work by Croskerry et al. [10] highlighted the importance of bringing attention to the notion that healthcare providers are not immune to emotional influences, and must therefore focus on not allowing their emotional experiences to negatively influence the care they provide.

Within this review, patients were described as the most common trigger eliciting emotions and subsequently influencing patient safety processes and outcomes. Although one study did also identify hospital and system-level factors as triggers [15], these were not explored in patient safety processes. As well as patients, many other factors within the healthcare work environment were identified in the first stage of this review as influencing the emotions staff experienced. However, how emotional responses to such triggers affect patient safety processes and outcomes is currently unclear and warrants further research. The studies reviewed here focused on the intrapersonal effects of emotion. Researchers have recently highlighted the need for further work to understand the social aspects of emotion [45–47]. The Emotions as Social Information (EASI) model [45] posits that many of our decisions and actions cannot be explained solely by individual thought processes, but are often due to social interaction which involves observing and responding to the emotional displays of others, providing a potentially useful framework for further exploration.

Workplace violence and patient aggression were identified in this review as triggers of emotions in the healthcare work environment. Research evidence suggests that gender plays a role in determining recipients who are subjected to workplace violence and the type of violence they may experience. Male healthcare staff report experiencing a higher prevalence of workplace violence compared to their female counterparts [48, 49]. Gender influenced the types of violence experienced by healthcare staff, where in general, female healthcare staff experienced more verbal violence, and male healthcare staff experienced more physical violence [48]. Different risk factors for workplace violence have been reported for males and females. For male healthcare staff, lower income levels and managers were at a higher risk of workplace violence, whereas longer working hours were associated with a higher risk of workplace violence among female healthcare staff [49].

As experiencing workplace violence and patient aggression have been found to have a negative impact on the delivery of patient care, this is a topic area that warrants further research. The majority of emotions identified in response to the triggers in this review were negative in nature. Within the few studies where positive emotions were mentioned, experiencing these as a result of a positive patient encounter was associated with increased interaction with patients, where healthcare staff perceived they were more engaged and provided expedited care [15, 16]. This finding is congruent with limited previous Research that suggests positive emotions may improve patient safety and patient care; positive affect led medical students to identify lung cancer in patients more quickly [50] and resulted in correctly diagnosing patients with liver disease sooner [51]. However, positive emotional responses may also have the opposite effect e.g. over-testing and over-treating patients, or reducing staff belief that the patient has a serious illness, resulting in adverse outcomes [16]. Greater understanding is required to articulate conditions and triggers of positive emotions and when these might support patient safety or cause harm [44].

### Limitations

There was heterogeneity within the included studies and the primary aim of most studies was not to answer the research questions posed here. To answer our research questions, it was necessary to include articles where the study aims addressed only one of the concepts of interest or where only limited associations between triggers of emotion or the emotions experienced in response to triggers and patient safety processes or outcomes were made. Moreover, it is important to recognise that there is likely to be some bias in the research literature, meaning that the triggers of emotion we identified from the current published research and the emotions experienced in response to these cannot be assumed to accurately represent the routine experiences of healthcare staff. Also worthy of note is that the majority of studies focus on negative triggers of emotions or the negative emotions experienced which may also lead to reporting bias. We acknowledge that we did not search studies before the year 2000.

### Implications for future research and practice

The triggers of emotion and types of emotion experienced that have been identified in this review can be used as a framework for further work examining the role of emotion in patient safety. Developing validated measures of the triggers of emotions, and the types of emotions experienced by healthcare staff in the work environment will facilitate this and is urgently needed. The findings also suggest that particular types of emotion were more frequently experienced in response to particular categories of triggers and that healthcare staff’s experiences of negative emotions have negative effects on patient care and ultimately, patient safety. This provides a basis for developing and tailoring strategies, interventions, and support mechanisms for dealing with either short-term or long-term consequences, and regulation of emotions in the healthcare work environment. For example, healthcare staff can be offered some time out from their clinical duties to take a brief pause when immediate and short-term emotional reactions are experienced. They may also be provided with one-to-one peer support to help healthcare staff experience a more reflective, self-conscious emotional response. It also highlights the possibility of preparing healthcare staff for likely emotional reactions in particular clinical situations to assist them in being more mindful of the possible impact on the safety of the care they provide. The limited research currently available suggests that emotions influence patient safety processes/outcomes. Further research is needed to explore this relationship further. For example, studies that focused exclusively on more amorphous emotional concepts like burnout were excluded. However, in some of the included studies, these longer-term emotional responses were identified in addition to the immediate, short-term, and reflective emotions. Further research needs to explore longer-term emotional responses such as PTSD, burnout, and work satisfaction, the associated triggers, and the impact on patient safety.

It is important to raise awareness of the potential impact of emotional triggers and the emotions experienced in response to these on patient safety through training and education for healthcare staff. As suggested by previous authors, we recommend that emotional awareness and regulation skills, both of which can be developed and enhanced using emotional intelligence training interventions [52, 53] are included in healthcare staff training [44, 54–56]. Future work should also distinguish between specific types of emotional responses rather than broadly classifying these as negative and positive, and explore how these influence patient safety. The findings also have potential implications for health equity given that the evidence indicates certain types of patients (e.g. angry and mental health patients) are more likely to provoke negative emotions, and such emotions can result in a negative impact on patient care and safety. This may suggest that such patient groups may receive poorer quality of care due to social factors beyond their control and is an area that requires further research.

## Conclusions

Healthcare staff are exposed to many emotional triggers within their work environment including patient safety events, traumatic events, work overload, workplace toxicity, lack of supervisory support, and patient and family factors. In response, healthcare staff experience emotions ranging from anger and guilt to longer-term burnout and PTSD symptoms. Both triggers and the emotional responses to these are perceived to negatively impact patient care and safety, although robust empirical evidence is lacking.

### Supplementary Information

**Supplementary Material 1.**

**Supplementary Material 2.**

**Supplementary Material 3.**

## Data Availability

The datasets used and/or analysed during the current study are available from the corresponding author on reasonable request.
