# Supplementary material for: A systematic review of workplace triggers of emotions in the healthcare environment, the emotions experienced, and the impact on patient safety
Source: BMC Health Serv Res. 2024 May 9;24:603. doi: 10.1186/s12913-024-11011-1 (PMC11080227; doi:10.1186/s12913-024-11011-1)
Supplement: Supplementary file 1 — Supplementary Material 1. [file 12913_2024_11011_MOESM1_ESM.docx]

Appendix 1: MEDLINE search terms

Concept 1: Adverse events and patient safety

1. Adverse event*
2. Incident*
3. Medical error* [MESH]
4. Hospital mortal*
5. Patient safety [MESH]
6. Medication error [MESH]
7. Safety metric*
8. Unsafe healthcare
9. Patient harm*
10. Patient incident*
11. Diagnostic error*
12. Near failure*
13. Readmission*
14. Safety incident*
15. Safety outcome*
16. Safety data
17. Incident report*
18. Patient safety outcome*
19. Healthcare safety
20. Harm*
21. Never event*
22. Serious incident*
23. Medical error*
24. Prescribing error*
25. Undesirable event*
26. Unsafe care experience*
27. Adverse drug event*
28. Unsafe practice*
29. Healthcare error*

Concept 2: Emotion

Emotion[MESH term]

1. Emoti*
2. Feel*
3. Fear*
4. Anger*
5. Disgust*
6. Sad*
7. Happ*
8. Surpris*

Concept 3: Healthcare professional

1. Healthcare professional*Health personnel [MESH]
2. Healthcare staff*
3. Nurse*
4. Nursing staff*
5. Physician*
6. Health professional*
7. Resident*
8. Healthcare team*
9. Healthcare employee*
10. Surgeon*
11. Doctor*
12. GP*
13. Registrar*
14. Consultant*
15. Allied health personnel [MESH]
16. Allied health worker*
17. Medical staff
18. Clinical team*
19. Medical team*
20. Midwi*
21. Clinician*
22. Health provider*
23. Healthcare provider*
24. Healthcare worker*
