# Supplementary material for: A systematic review of workplace triggers of emotions in the healthcare environment, the emotions experienced, and the impact on patient safety
Source: BMC Health Serv Res. 2024 May 9;24:603. doi: 10.1186/s12913-024-11011-1 (PMC11080227; doi:10.1186/s12913-024-11011-1)
Supplement: Supplementary file 2 — Supplementary Material 2. [file 12913_2024_11011_MOESM2_ESM.docx]

Appendix 2: Detailed process for the categorisation of triggers of emotions, and emotions experienced

***Research Question 1: What triggers emotions in the healthcare work environment?***

*Categorising the triggers:* The triggers of emotion were extracted directly from the 90 studies using the author's own terminology. Due to some repetition across studies, this resulted in 67 triggers being identified. The first step in the process of categorisation involved a team of 12 patient safety researchers using an approach similar to content analysis, and linking common terms (e.g. adverse event, medical error, nursing error) independently. A large group discussion with all 12 patient safety researchers followed this, resulting in 19 categories. Each patient safety researcher shared their categorisations with justifications, and any disagreements were resolved through discussions. The 19 categories were further refined by linking categories with a shared theme e.g. those that related to interactions with patients and family. This was an iterative process, completed individually by team members, followed by discussions between the team. We then presented these categories to a large group of patient safety researchers (n=16) and the items within each one, at an inter-disciplinary meeting and their face validity was confirmed. In-depth discussions took place and any suggested changes were discussed in detail and where appropriate, incorporated into the final version of the categories.

***Research Question 2: What are the emotions experienced in response to these triggers?***

*Categorising emotions:* The emotion literature is complex, but three key factors are frequently used to distinguish emotions: viscerality - the physicality and instinctiveness of the response, the level of reflection (impact of cognition), the extent to which they reflect how we think others see us (self-consciousness) and length of effect (short term/fleeting – chronic). Of course, these three factors are interrelated with, for example, visceral responses being short-lived and with little conscious awareness. Here, we aimed to distinguish between the different types of emotional responses and categorise the variations accordingly. A team of 6 patient safety researchers, including an emotion expert were involved in developing the categories for the different types of emotions. This process was initially conducted independently by each of the researchers and a group discussion followed. This resulted in a refined classification and was presented to our wider patient safety centre team, after which final iterations were made. Sub-categories within these four higher-order categories were then based on the author's descriptions of discrete emotions and the use of emotion terms within their work.
